# Dr. John Thad. Johnson

**Published:** 1884-12

**Authors:** 


### DR. JOHN THAD. JOHNSON.

Dr. Johnson, of whom mention was made some months ago in THE JOURNAL, as contemplating removal to California, left Atlanta November 12th for his new field. It has been our good fortune to know Dr. Johnson intimately for a number of years, and we feel safe in saying that no young man has ever risen more rapidly in the medical world than he. Dr. Johnson graduated in medicine in 1868 in Baltimore, and began the practice of medicine soon thereafter in this city. In a short time he was elected Demonstrator of Anatomy in the Atlanta Medical College. He served in this position until he was chosen Professor of Anatomy in the same College, and also elected Dean of the Faculty. He filled the chair of Anatomy to the entire satisfaction of the Board of Trustees, and his administration was eminently successful. In 1878 he was elected President of the Medical Association of Georgia.

In 1880, Dr. Johnson resigned the office of Dean and the professorship of Anatomy in the Atlanta Medical College, and accepted the professorship of Surgery in the Southern Medical College. His thorough knowledge of anatomy rendered him peculiarly fitted to fill this important chair. As a teacher and surgeon he is justly popular, having made many important operations in this city and State. He has made many valuable contributions to medical literature.

In 1872 he was married to Miss Anna Banks, daughter of the late Dr. J. T. Banks, of Griffin, Ga., who was one of the most prominent physicians in this State. Fortunate in his choice of a wife, he owes much that he has achieved to her influence. Cultivated, intelligent and refined, she has appreciated his efforts to attain distinction in his profession, while he has been constantly blessed by her cheerful spirits and affectionate disposition.

Socially, Dr. Johnson stands deservedly high. We regret exceedingly that he has decided to make his home elsewhere, and we voice the sentiment of hundreds of the best people in Atlanta when we wish for him and his family a safe trip and a pleasant sojourn in their new home.

